# Molecular surveillance and genomic characterization of enterovirus D68 in southern Portugal, 2024–2025

**DOI:** 10.1007/s00705-026-06655-9

**Published:** 2026-05-22

**Authors:** Sérgio Santos-Silva, Guilherme Moreira, André Palma, Soraia Rodrigues, Margarida Simões, Raquel Guerreiro, Maria S.J. Nascimento, João R. Mesquita

**Affiliations:** 1 LAQV, REQUIMTE, Department of Chemistry and Biochemistry, Faculty of Sciences, University of Porto, Porto, Portugal; 2 School of Medicine and Biomedical Sciences (ICBAS), University of Porto, Porto, Portugal; 3 Serviço de Patologia Clínica, Unidade Local de Saúde do Algarve – Unidade Hospitalar de Faro, Faro, Portugal; 4 Department Veterinary Medicine, Comprehensive Health Research Centre, University of Évora, School of Science and Technology, Évora, Portugal; 5 Faculty of Pharmacy, University of Porto (FFUP), Porto, Portugal

## Abstract

Enterovirus D68 (EV-D68) is a re-emerging respiratory pathogen that is increasingly associated with severe respiratory disease and occasional neurological complications, yet its circulation in Portugal remains poorly documented. In the present study, we investigated the presence of EV-D68 in respiratory samples collected in southern Portugal (Algarve region) from October 2024 to February 2025. Screening of 150 respiratory samples from patients (115 from children and 35 from adults), all previously positive for rhinovirus/enterovirus, revealed eight EV-D68–positive cases, corresponding to a detection rate of 5.44%, spanning a broad age range from infancy to the elderly. Clinical manifestations ranged from mild respiratory illness to severe conditions, including pneumonia and acute respiratory distress syndrome. Neurological manifestations, including complex febrile seizures and encephalomyelitis, were also observed in EV-D68–positive patients. Some cases involved coinfections, while others presented with EV-D68 as the sole detected respiratory pathogen. Genomic characterization from one isolate revealed a VP1 fragment belonging to clade B3, and a complete genome from another isolate clustered within clade A2. Phylogenetic analysis confirmed clustering with contemporary European isolates. These findings provide preliminary evidence of EV-D68 detection in southern Portugal and document the presence of clades A2 and B3 among the characterized samples. The results illustrate the heterogeneous clinical presentations observed in affected patients and highlight the value of continued molecular monitoring and genomic characterization of EV-D68.

**Supplementary Information:**

The online version contains supplementary material available at 10.1007/s00705-026-06655-9.

## Introduction

Enterovirus D68 (EV-D68) is a re-emerging non-polio enterovirus in the family *Picornaviridae*, first isolated in 1962, that primarily causes respiratory illness yet has increasingly been associated with more severe lower respiratory tract disease and, in rare cases, neurologic complications such as acute flaccid myelitis (AFM) [1].

Historically, EV-D68 caused sporadic cases or small clusters of acute respiratory illness but from around 2014 onward, several large outbreaks of acute respiratory illness have been well documented in North America [2–4] and Europe [5–7]. These outbreaks revealed not only elevated numbers of respiratory disease, often among children, but also occasional neurological manifestations, raising concern about viral evolution, diversity, and surveillance gaps [8, 9].

Molecular studies have improved the understanding of EV-D68 genotype and clade structure, identifying four main clades (A, B, C, and D), some of which further subdivide into subclades (A1, A2, B1, B2, B3, D1, D2), with subclades such as B3 being especially prominent in recent years [10]. Complete genome sequencing is still relatively rare in many settings, limiting resolution of viral evolution, transmission dynamics, and potential recombination or mutation events outside highly variable regions like VP1 [11]. The VP1 region encodes the major capsid protein and is the principal genomic target used for enterovirus classification, molecular typing, and phylogenetic analysis due to its genetic variability and antigenic relevance [12].

Recent work by the European Non-Polio Enterovirus Network (ENPEN) has shown a marked upsurge of EV-D68 in Europe during the fall–winter 2021–2022 season, with severe acute respiratory distress commonly observed, often presenting with fever and, in some cases, neurological complications, including AFM diagnosed in young children, alongside the emergence of B3-derived lineages [10, 13]. Moreover, sustained circulation of EV-D68 and evolution of these B3-derived lineages (including novel amino acid substitutions) demonstrate the rapid adaptive capacity of EV-D68, even during periods of presumed low viral activity, and have once again been documented across Europe [10], in the United States of America [14] and parts of Asia [15–17]. These findings point to both ongoing genetic diversification and the need for improved, harmonized detection and surveillance [10].

In Portugal, the circulation or genetic diversity of EV-D68 remains largely uncharacterized. To address this gap, we conducted a study in southern Portugal (Algarve region) from October 2024 to February 2025, screening nasopharyngeal samples from children and adults who presented to the emergency department or required hospitalization with acute and severe respiratory infections and tested positive for rhinovirus/enterovirus (RV/EV), in order to detect the presence of EV-D68. We also aimed to characterize the EV-D68 isolates identified at the genomic level by performing amplification of the VP1 region and, when possible, attempting whole genome sequencing.

This study contributes additional data on EV-D68 detection and genetic characterization in Portugal through phylogenetic analysis of the identified isolates.

## Materials and methods

### Sample collection

A total of 150 nasopharyngeal samples were collected from 115 children and 35 adults who attended the emergency department or were hospitalized at Unidade Hospitalar de Faro (Algarve, Portugal) with acute and severe respiratory infections between October 2024 and February 2025. All samples tested positive for Rhinovirus/Enterovirus (RV/EV) using the BioFire^®^ FilmArray^®^ Respiratory 2.1 Panel (BioMérieux). All the nasopharyngeal swab samples collected in viral transport medium were stored at − 80 °C until further processing. Co-infecting respiratory viruses and other pathogens were reported by the hospital diagnostic team based on routine clinical testing (including BioFire^®^ FilmArray^®^ Respiratory 2.1 Panel (BioMérieux) where available), and bacterial testing was performed only when clinically indicated and extracted from patient records.

### Nucleic acid extraction for EV-D68 detection

Nucleic acids were extracted from 200 µL of each of the 150 original nasopharyngeal swab samples using the QIAamp Viral Mini Kit (Qiagen, Hilden, Germany) on the QIAcube^®^ automated platform (Qiagen), following the instructions from the manufacturer. Total nucleic acids were eluted in RNase-free water and stored at − 80 °C until further processing.

### Detection of EV-D68 and genomic characterization

EV-D68 RNA screening was performed in all 150 RV/EV-positive samples using a real-time RT-PCR (RT-qPCR) assay targeting a ~ 94 bp fragment in the VP1 region (NU assay) [18]. RT-qPCR reactions were performed on a CFX Connect Real-Time PCR Detection System (Bio-Rad, Hercules, CA, USA) using the Xpert OneStep Fast Probe kit (GRiSP^®^, Porto, Portugal), according to the manufacturer’s instructions. Thermal cycling included an initial reverse transcription step at 50 °C for 15 min, followed by reverse transcriptase inactivation and cDNA denaturation at 95 °C for 5 min. Amplification was then carried out over 40 cycles, with denaturation at 95 °C for 5 s and annealing/extension at 55 °C for 20 s. Data were analyzed using CFX Maestro software version 4.0.2325.0418 (Bio-Rad, Hercules, CA, USA).

Further EV-D68 genomic characterization was performed by nested PCR amplification of the VP1 region (~ 590 bp) in all EV-D68–positive samples using the set of primers AN1019, AN1014 and AN1021, AN1022 previously described [18]. All PCR reactions were performed on a T100 thermocycler (Bio-Rad, Hercules, CA, USA). The first PCR round was carried out using the Xpert One-Step RT-PCR kit (GriSP^®^, Porto, Portugal), followed by the second round with the Xpert Fast Hotstart Mastermix 2× with dye (GriSP^®^, Porto, Portugal). Thermal cycling for the first round included cDNA synthesis at 45 °C for 15 min, initial denaturation at 95 °C for 3 min, then 40 cycles of 95 °C (denaturation) for 10 s, 60 °C for 10 s (annealing), and 72 °C for 15 s (extension), with a final extension at 72 °C for 10 min. The second round consisted of an initial denaturation at 95 °C for 3 min, followed by 40 cycles of 95 °C (denaturation) for 15 s, 52 °C for 15 s (annealing), and 72 °C for 2 s (extension), concluding with a final extension at 72 °C for 10 min.

The PCR products were visualized by electrophoresis on a 1% agarose gel stained with Xpert Green Safe DNA gel dye (GriSP^®^, Porto, Portugal) and run at 120 V for 30 min. The results were confirmed using a UV transilluminator.

Amplicons of the expected size were purified using the GRS PCR & Gel Band Purification Kit (GriSP^®^, Porto, Portugal). Purified products were then subjected to bidirectional Sanger sequencing using the appropriate specific internal primers for the target gene.

### Whole-genome sequencing

Sequence-independent single-primer amplification (SISPA) was performed on positive RT-qPCR samples with a previously described protocol [19]. The cDNA generated through SISPA was sequenced on an Oxford Nanopore Technologies (ONT) PromethION 24 platform using an R10.4.1 flow cell. Libraries were prepared with the Native Barcoding Kit 96 V14 (SQK-NBD114.96).

### Bioinformatic analysis

Raw FASTQ reads were basecalled in super-accurate mode using ont-dorado-for-promethion v.7.4.12, with a minimum Q-score threshold of 10, and adapters and barcodes trimmed via MinKNOW. Initial read quality was assessed with NanoPlot v.1.43.0 [20]. Sequencing adapters and barcodes were further removed using Porechop v.0.2.4 [21], and reads were filtered with NanoFilt v.2.8.0 [20] to retain only those with an average quality score of at least 10.

Filtered sequencing reads were first screened to remove host- and contaminant-derived sequences. This was done by aligning the reads to reference FASTA files representing the host genome and potential contaminants using Minimap2 [22]. Reads that failed to align (i.e., unmapped reads) were kept for downstream analyses. These unmapped reads were taxonomically classified with Kraken2 [23] against the RefSeq viral database retrieved on 1 September 2025 [24], to identify candidate viral sequences. Reads classified as EV-D68 were then extracted using the extract_kraken_reads.py script [23] which retrieves reads based on Kraken2 classification results.

The extracted EV-D68 reads were subsequently mapped to a reference EV-D68 genome. The reference genome was indexed with Minimap2 (v2.30) [22], which is optimized for long-read sequencing data such as Oxford Nanopore. Reads were aligned with the map-ont preset, generating a SAM file that was converted to a sorted and indexed BAM file using Samtools (v1.22) [25], for efficient handling. Genome-wide coverage was then calculated from the BAM file with Pysam (v0.23.3) [26]. Per-base coverage was computed by iterating through pileup columns and the resulting coverage profiles were generated using an in-house Python script [27], using Matplotlib [28].

A consensus sequence was assembled from the aligned reads in regions with sufficient depth and overlap. This consensus sequence was queried against the NCBI nucleotide database using BLASTn [29] to validate viral identity and assess sequence similarity.

The resulting bidirectional Sanger sequences were edited and aligned using BioEdit v7.1.9 and compared with sequences available in the NCBI GenBank nucleotide database, retrieved on 1 September 2025 (http://blast.ncbi.nlm.nih.gov/Blast).

### Phylogenetic analysis

The sequences obtained in this study were compared with EV-D68 reference sequences retrieved from the Nextstrain EV-D68 dataset (accessed on 1 September 2025) for phylogenetic analysis [32]. Multiple sequence alignment was conducted using MAFFT software version 7.407 [30]. Maximum-likelihood phylogenetic trees were then inferred with IQ-TREE, employing automatic model selection and 1,000 bootstrap replicates to assess node support. Trees were visualized and annotated using the Interactive Tree Of Life (iTOL) platform.

### Recombination analysis

To assess potential recombination events, the complete EV-D68 genome recovered in this study was analyzed together with representative complete EV-D68 reference genomes from different clades/subclades. Multiple sequence alignment was performed using MAFFT software version 7.407 [30]. Phylogenetic trees were independently inferred for the 5’UTR, P1, P2, and P3 genomic regions using IQ-TREE software version 7.407 [30], under the best-fit substitution model. Putative recombination events were further investigated using Recombination Detection Program version 4 (RDP4), applying the methods RDP, GENECONV, BootScan, MaxChi, Chimaera, SiScan, and 3Seq with default parameters. To improve the reliability of recombination detection, several algorithms are typically applied simultaneously, and recombination signals are generally considered meaningful when they are supported by more than four methods. Similarity plot analyses was additionally performed using SimPlot++ [31].

## Results

### Demographic and clinical characteristics of EV/RV-positive patients

From the 150 patients studied, all shared a common finding: they were all positive for RV/EV. The study population consisted of 115 children and 35 adults. The children’s ages ranged from 1-week-old to 17-year-old, with a median age of 12-month-old and a mean age of approximately 2-year-old and 7-month-old. The group was predominantly male (*n* = 75). The adult’s age ranged from 20 to 100-year-old with a median age of 58-year-old, and a mean age of approximately 60-year-old and 2-month-old. From this group 19 were males and 16 females.

The most frequent clinical presentations in children at the hospital admission included fever (*n* = 39), cough (*n* = 14), bronchiolitis (*n* = 7), broncospasm (*n* = 5) and asthma (*n* = 5). In addition, more severe clinical manifestations were documented, including acute respiratory distress syndrome (ARDS) (*n* = 1), complex febrile seizures (*n* = 1), and other serious conditions such as autoimmune encephalitis (*n* = 1). For adults the most frequent clinical presentation at the hospital admission included pneumonia (*n* = 5), fever (*n* = 3), bronchiolitis (*n* = 1), cough (*n* = 1), difficulty breathing (*n* = 2), and asthma or chronic lung disease (*n* = *2*). Furthermore, more severe manifestations were documented, including hypocalcemia (*n* = 1), diabetic ketoacidosis (*n* = 1), neutropenia with fever (*n* = 1), and immunosuppression (*n* = 2) in the adult’s group.

In addition to testing positive for RV/EV, co-infections with other respiratory viruses were identified in some patients, including adenovirus (*n* = 22), respiratory syncytial virus (RSV) (*n* = 8), parainfluenza viruses types 1, 2, and 3 (*n* = 8), influenza A viruses (H1 and H3) (*n* = 5), influenza B virus (*n* = 4), coronaviruses NL63 (*n* = 3) and OC43 (*n* = 3), human metapneumovirus (*n* = 3), and SARS-CoV-2 (*n* = 1).

### Demographic and clinical characteristics of EV-D68 positive patients

Among the 150 RV/EV-positive samples analyzed for EV-D68, eight tested positive, five were from children and three from adults (Table 1).

**Table 1:** Clinical and virological characteristics of the eight EV-D68–positive patients, including age, sex, clinical presentation, coinfections, sample collection date, and viral clade/subclade when available

Sample ID	Age	Sex	Clinical presentation	Coinfections	Ct value	Month of isolation	Clade/subclade detected (Accession number)
1	8 months	Male	ARDS	-	31.83	November 2024	-
2	1 year	Male	Tachypnea, difficulty breathing and cough	Adenovirus	21.50	December 2025	B/B3 (PX277134)
3	2 years	Male	Global polypnea, bronchospasm, persistent cough	Adenovirus	24.97	December 2024	-
4	2 years	Female	Complex febrile seizure	Adenovirus and RSV	30.73	January 2025	-
5	8 years	Male	Encephalomyelitis, persistent fever, global respiratory failure requiring invasive ventilation	-	22.78	December 2024	-
6	68 years	Male	Pneumonia, global respiratory failure	-	19.46	January 2025	A/A2 (PV418226)
7	80 years	Female	Severe fatigue and loss of strength in the lower limbs	-	31.73	November 2024	-
8	95 years	Female	Tracheobronchitis, hypoxemic respiratory failure	-	15.10	November 2024	-

*ARDS * Acute respiratory distress syndrome, *RSV * Respiratory syncytial virus

The EV-D68 positive children were aged 8-month-old, 1-year-old, 2-year-old, and 8-year-old, comprising four males and one female. Co-infections of EV-D68 and adenovirus were observed in three cases, one of which was also infected with RSV and presented with complex febrile seizures. Noteworthy, the two children with the most severe clinical conditions, one with ARDS and the other with global respiratory failure requiring invasive ventilation, periods of drowsiness, and encephalomyelitis, did not present detectable viral co-infections, and EV-D68 was the only respiratory virus identified. However, a direct causal relationship cannot be definitively established.

The three adults who tested positive for EV-D68 were 68, 80, and 95-year-old, and included two males and one female. No co-infections were identified in this group, and the clinical conditions included tracheobronchitis, pneumonia, global respiratory failure, with one case reporting weakness of the lower limbs.

### Analysis of EV-D68 Strains

In order to characterize EV-D68 at the genomic level, nested PCR amplification of the VP1 region (~ 590 bp) was performed on all the eight EV-D68–positive samples.

Only one sample, obtained from a 2-year-old child yielded a VP1 gene fragment suitable for analysis. The sequence has been deposited in GenBank under the accession number PX277134 (Table 1).

Using the SISPA protocol, we successfully recovered a complete EV-D68 genome out of the eight EV-D68 positive samples. The genome obtained from a 68-year-old patient has been deposited in the GenBank database under accession number PV418226 (Table 1).

The two successfully characterized samples had relatively low RT-qPCR Ct values (21.50 for the partial VP1 sequence and 19.46 for the complete genome), suggesting that higher viral RNA levels may have contributed to sequencing success.

Depth-of-coverage analysis of the assembled genome indicated high and relatively uniform coverage across most of the genome (Supplementary Fig. 1). A graphical representation of the coverage confirmed sufficient read depth to ensure high confidence in consensus sequence generation.

**Fig. 1:**
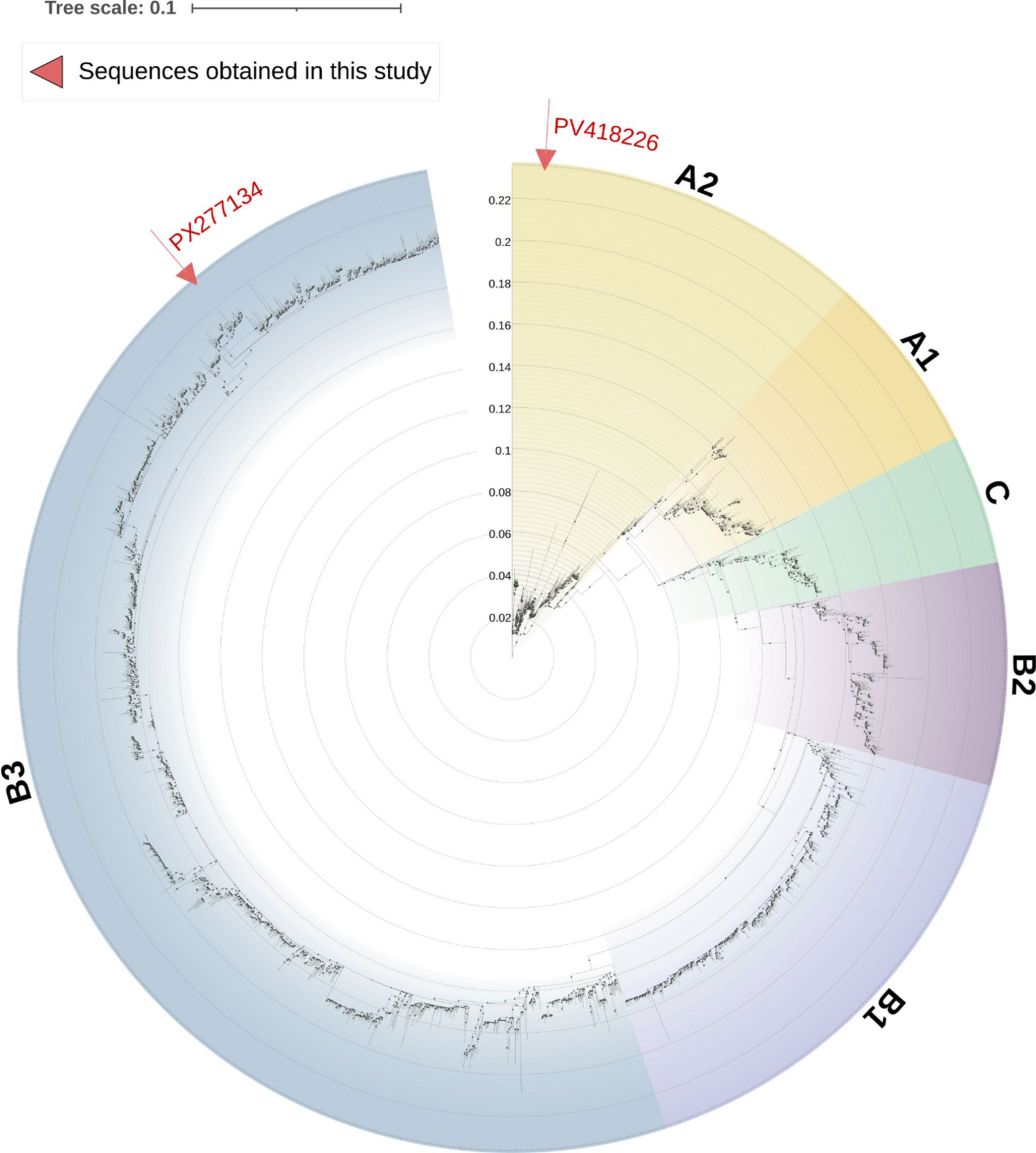
Maximum-likelihood phylogenetic tree of EV-D68 based on the full VP1 gene. The tree was inferred using the TIM + F+R4 substitution model (Transition model with empirical base frequencies and a FreeRate model of rate heterogeneity using four discrete rate categories), selected as the best-fitting model according to the Bayesian Information Criterion (BIC). All available Nextstrain VP1 sequences at the time of retrieval were included to provide a comprehensive overview. Node support values represent bootstrap percentages based on 1,000 replicates. The full VP1 sequences obtained in this study (PX277134 and PV418226) are highlighted by red arrows

**Fig. 2:**
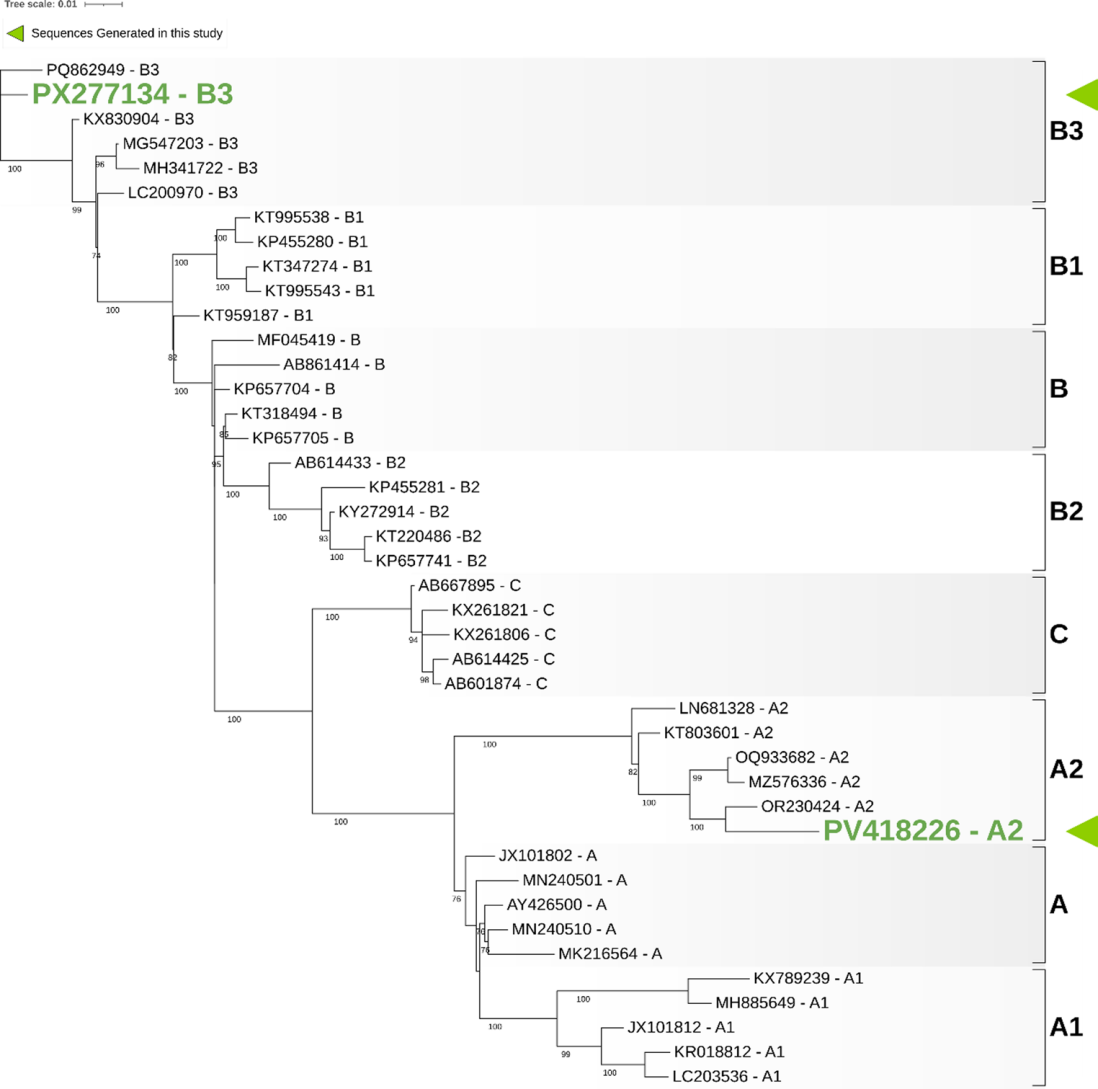
Maximum-likelihood phylogenetic tree of EV-D68 based on the full VP1 gene. The tree was inferred using the TN + F+G4 substitution model (Tamura-Nei model with empirical base frequencies and a Gamma model of rate heterogeneity using four discrete rate categories), selected as the best-fitting model according to the Bayesian Information Criterion (BIC). The dataset included five representative VP1 sequences per clade/subclade available at the time of retrieval. Node support values correspond to bootstrap percentages derived from 1,000 replicates. Accession sequence numbers are shown together with clade/subclade designations, and the VP1 sequences obtained in this study (PX277134 and PV418226) are highlighted in green bold

**Fig. 3:**
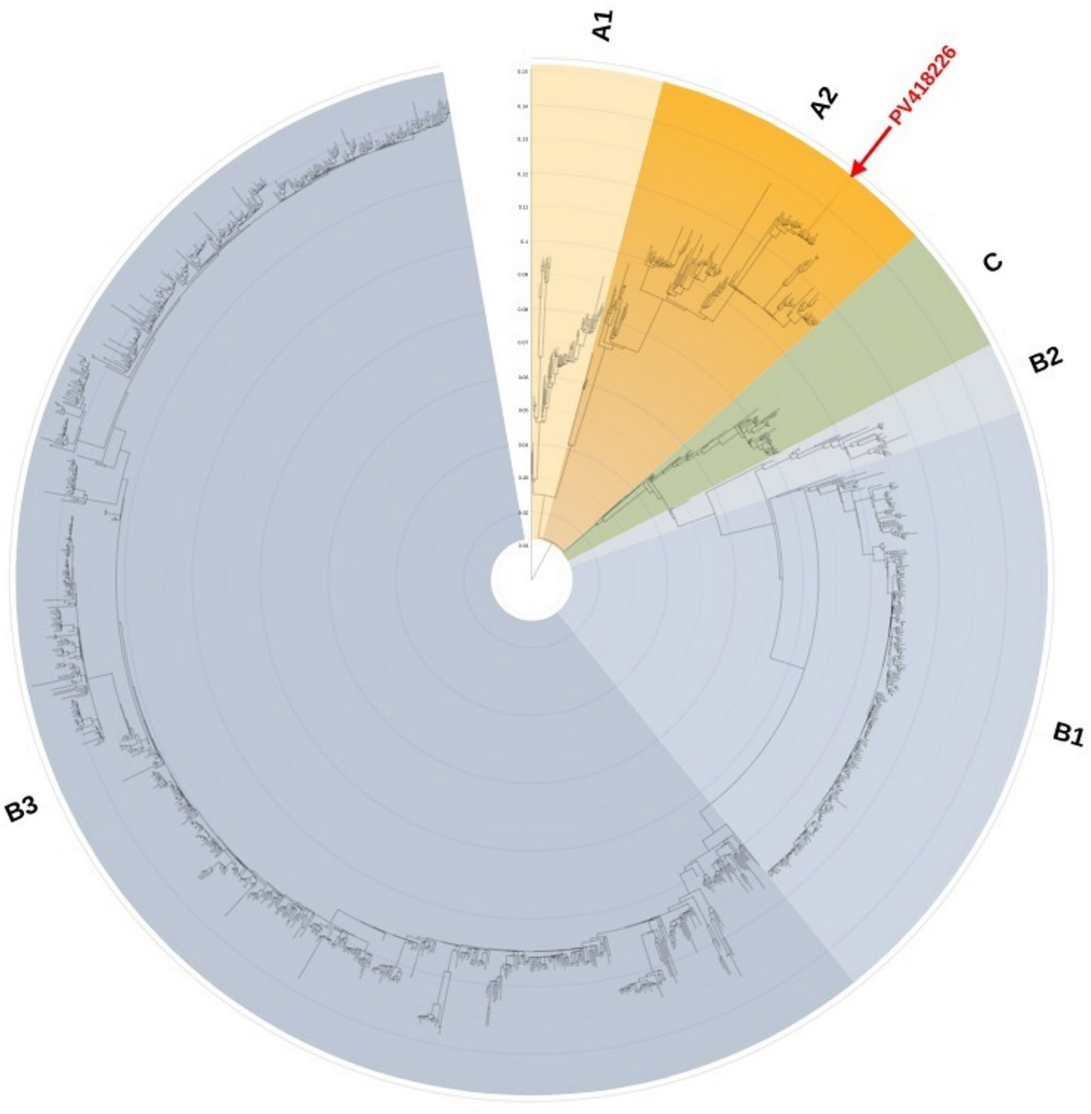
Maximum-likelihood phylogenetic tree of EV-D68 based on the full genome. The tree was inferred using theTIM + F+I+R6 substitution model (Transition model with empirical base frequencies and an Invar+FreeRate model of rate heterogeneity using six discrete rate categories), selected as the best-fitting model according to the Bayesian Information Criterion (BIC). Node support values indicate bootstrap percentages based on 1,000 replicates. All complete EV-D68 genomes available in the Nextstrain dataset at the time of retrieval were included. The full genome PV418226 obtained in this study is highlighted by a red arrow

Three phylogenetic trees were constructed to determine the evolutionary placement of the obtained sequences. First, a tree based on the complete VP1 gene was constructed using all available reference sequences from Nextstrain [32], allowing comparison with a broad set of isolates (Fig. 1). In this analysis, the VP1 gene fragment obtained from the child (PX277134) clustered within clade B, subclade B3, while the VP1 gene from the full genome obtained from the 68-year-old patient (PV418226) clustered within clade A, subclade A2. Second, a smaller VP1-based tree was generated using a subset of representative sequences, including five sequences from each clade and subclade available in Nextstrain [32], to provide a simplified overview of lineage relationships (Fig. 2). This analysis confirmed that our sequences belong to the same lineage as shown in the Fig. 1 phylogenetic tree. Finally, a full-genome phylogenic tree was constructed incorporating all available complete EV-D68 genomes from Nextstrain [32], alongside the full genome recovered in this study PV418226 (Fig. 3). The full genome clustered within clade A, subclade A2, along with other European isolates, such was the case with the VP1-only analyses. BLAST analysis of this full genome indicated that it shared 99.57% identity with an EV-D68 full genome respiratory sample isolated from a human in France (PQ612569), and 99.33–99.39% identity with EV-D68 full genome respiratory samples isolated from humans in the USA (PV624835, PV178657), respectively.

No supported recombination events were detected in the recovered complete genome by RDP4 analysis (Supplementary Fig. 2). Consistently, phylogenetic inference of the 5′UTR, P1, P2, and P3 genomic regions showed stable clustering within clade A2 (Supplementary Fig. 3), and similarity plot analysis did not reveal evidence of mosaic genome structure (Supplementary Fig. 4).

## Discussion

EV-D68, a re-emerging respiratory pathogen increasingly linked to severe respiratory disease and neurological complications [1, 33], has shown genetic diversification and widespread circulation across Europe [10, 13], yet its presence in Portugal remains poorly documented. The present study reports the detection and genomic characterization of EV-D68 in respiratory samples collected in southern Portugal, contributing to the limited data available on EV-D68 in Portugal. Phylogenetic analysis of the characterized sequences provided additional genomic data on EV-D68 detected in this region of the Iberian Peninsula and their relationship with circulating European lineages.

Of the 150 patients studied, all of whom were RV/EV-positive, eight tested positive for EV-D68, corresponding to a detection rate of 5.44% in nasopharyngeal samples. The age of patients infected with EV-D68 ranged from 8-month-old to 95-year-old, with both sexes represented. Clinical presentations were heterogeneous for both pediatric and adult patients.

Among children infected with EV-D68, clinical manifestations included acute respiratory distress syndrome-ARDS (8-month-old, male), tachypnea with difficulty breathing and cough (1-year-old, male), global polypnea with bronchospasm and persistent cough (2-year-old, male), complex febrile seizure (2-year-old, female), and encephalomyelitis with persistent fever and global respiratory failure requiring invasive ventilation (8-year-old, male). Among adults, presentations included pneumonia with global respiratory failure (68-year-old, male), severe fatigue and loss of strength in the lower limbs (80-year-old, female), and tracheobronchitis with hypoxemic respiratory failure (95-year-old, female). This is in line with what has been described in other studies where several international reports confirm that EV-D68 can cause a spectrum of disease ranging from mild illness to severe respiratory failure, in both children and adults, especially in the presence of respiratory comorbidities, such as asthma or chronic lung disease [13, 34–37]. In children, infection is often associated with respiratory symptoms such as cough, wheezing, shortness of breath, and fever, and it may progress to the need for intensive support, including mechanical ventilation, especially in patients with preexisting asthma or underlying lung disease [13, 35, 38–40]. Neurological complications, such as AFM, although rare, have been documented, reinforcing the neurotropic potential of the virus [41–43]. In adults, the clinical presentation is also predominantly respiratory, ranging from mild symptoms to severe respiratory failure, with greater severity observed in older adults and in those with comorbidities such as heart disease, asthma, and chronic obstructive pulmonary disease [34, 37]. Symptoms such as cough, shortness of breath, wheezing, chest pain, and fever are common, and hospitalization may be necessary, especially in patients with risk factors. Cases of neurological involvement in adults are rare, but have been reported [37, 42]. Given the small number of EV-D68-positive cases and the presence of coinfections, no causal relationship between EV-D68 infection and clinical severity can be inferred in the present study. Future studies including larger cohorts and systematic virological testing will be necessary to better assess potential associations between EV-D68 infection and clinical outcomes.

Coinfections with adenovirus and/or RSV were detected among pediatric patients, whereas no viral coinfections were identified in EV-D68–positive adults.

Molecular characterization was performed on two EV-D68 isolates, one from a 2-year-old patient and the other from a 68-year-old patient, revealing distinct genetic profiles. The VP1 gene fragment isolated from the sampled child patient clustered with subclade B3, while the full genome isolated from the adult was classified as subclade A2. According to Nextstrain representation of isolates over the past six years, the A2 subclade has been predominantly detected in Spain, France, and Italy, with the latter being the closest related sequences. Furthermore, only one sample yielded a VP1 amplicon suitable for Sanger sequencing, likely due to differences in RNA quality and amplification efficiency among samples. In contrast, the SISPA-based approach enabled recovery of a complete genome, supporting its utility for EV-D68 genomic characterization. Additional recombination analyses did not detect evidence of recombination in any region of the recovered A2 genome.

These results provide preliminary phylogenetic characterization of EV-D68 detected in Portugal, showing consistency with previous studies indicating that, since 2017, only genotypes B3 and A2/D2 have continued to circulate in Europe, as well as in Asia and the United States [1, 10, 44]. Moreover, the Algarve region in southern Portugal [45], as an international tourist destination, may contribute to the introduction and spread of diverse EV-D68 strains, highlighting the importance of continuous surveillance in areas with high population mobility. The identification of a full genome clustering in clade A2, together with a VP1 fragment from subclade B3 aligns with recent European trends where B3 has been the dominant lineage whilst A2 continues to be observed in circulation [33]. These findings indicate close genetic relatedness to recently reported European and North American A2 strains [33]. In 2018, European EV-D68 isolates showed distinct clusters in both B3 and A2, with different phylogenetic origins. Subclade B3 emerged mainly from U.S. and European lineages, whereas A2 was traced back to strains from East Asia [33]. Interestingly, subclade B3 has been primarily associated with pediatric infections (median age: 5-year-old), while A2 has been more common in adults (median age: 42-year-old) [46], a pattern that is consistent with the limited observations in our dataset. Complete genome and VP1 fragment analyses suggest the presence of these lineages in our setting, consistent with EV-D68 lineages previously reported in other European countries [33]. Future studies including larger numbers of complete EV-D68 genomes will be important to investigate mutation patterns, possible recombination events, and their epidemiological significance.

Although our dataset is too limited to analyze age or clinical severity associations, these findings highlight the need for further studies to better characterize the clinical spectrum of EV-D68 infection in Portugal.

When compared with other European studies, the 5.44% detection rate observed in our study falls within similar ranges, likely due to the use of respiratory samples, including remnant specimens that had previously tested positive for RV/EV. It should be noted that for the diagnosis of EV-D68, respiratory samples are considered the gold standard, however, in cases of neurological involvement, cerebrospinal fluid (CSF) analysis can also provide useful information, although viral detection in the CSF is rare [12, 47]. Moreover, the lack of standardized inclusion of EV-D68 in routine respiratory virus panels may contribute to the limited national-level data. For instance, large-scale surveillance during the 2021–2022 upsurge in Europe documented detection rates in respiratory samples ranging from below 5% in some hospital cohorts to over 10% [13]. Other surveys have also reported detection rates in respiratory or nasopharyngeal samples ranging from 2% to 5% in hospitalized children and exceeding 10% in pediatric outbreak settings [48, 49]. These data confirm that the 5.44% rate detected in the present study is consistent with the range reported in various European surveillance settings, some of which also included samples previously positive for RV/EV, as was the case in our study. However, because only samples previously testing positive for RV/EV were included, this detection rate reflects EV-D68 frequency within a pre-screened cohort rather than among all patients with respiratory infection. As such, this targeted sampling strategy may introduce selection bias and should not be interpreted as a population-level prevalence estimate. Furthermore, other surveillance studies have noted substantial variability across countries and seasons, often influenced by differences in sampling strategy, case mix, and testing intensity [33]. Although our dataset is limited in size and geographic scope, the measurable detection of EV-D68 in Portugal indicates ongoing viral circulation.

The antigenic evolution of EV-D68 remains a concern, particularly with amino acid substitutions in the VP1 loops and other surface-exposed protein regions [33]. European surveillance has demonstrated that these mutations can accumulate rapidly under immune pressure, potentially altering neutralization profiles and population susceptibility [50]. Future studies incorporating broader sequencing, epitope mapping, and serological assays will be essential to assess whether strains retrieved in Portugal show signs of immune escape or altered pathogenicity.

From a public health perspective, our findings carry several implications. First, the detection of EV-D68 in this study indicates its presence in southern Portugal during the study period, and support the need for continued surveillance being required to monitor its prevalence, genetic diversity, and potential clinical impact for both this specific region and the rest of the country. Second, expanded sequence-based monitoring could improve understanding of EV-D68 genetic diversity and lineage distribution in Portugal, since PCR detection alone does not provide the resolution needed to track introductions, clade shifts, or evolutionary dynamics. Finally, integration of molecular data with clinical and epidemiological information will be necessary to determine whether subclades A2 and B3 infections in Portugal show different severity or age distribution, as suggested in other European studies. Also, continuous collaborative data sharing with European partners is essential, given that EV-D68 shows rapid cross-border mixing, with migration rates between countries estimated at multiple introductions per year. This is particularly relevant for southern Portugal, including the Algarve region, which receives migratory flows from Africa and for which clinical and epidemiological surveillance data remain largely lacking.

In spite of these results, some limitations should be acknowledged. The present study was season restricted and geographically limited to the Algarve region, preventing assessment of national-level trends or longer-term evolutionary dynamics. In addition, only one VP1 sequence and one complete genome were recovered, limiting broader inferences regarding lineage circulation and age-related distribution patterns.

In conclusion, this study documents the detection of EV-D68 in respiratory samples collected in southern Portugal, including the detection of clades A2 and B3 among the characterized samples, which have also been detected circulating across Europe. These findings support the value of continued molecular monitoring and expanded genomic characterization, and integration of clinical and epidemiological data to better assess the potential burden and public health significance of EV-D68 in Portugal.

## Supplementary Information

Below is the link to the electronic supplementary material.

Supplementary Material 1 (DOCX 490 KB)

## Data Availability

The data that support the findings of this study are available from the corresponding author upon reasonable request.
