# Supplementary material for: Molecular surveillance and genomic characterization of enterovirus D68 in southern Portugal, 2024–2025
Source: Arch Virol. 2026 May 22;171(6):189. doi: 10.1007/s00705-026-06655-9 (PMC13194316; doi:10.1007/s00705-026-06655-9)
Supplement: Supplementary file 1 — Supplementary Material 1 (DOCX 490 KB) [file 705_2026_6655_MOESM1_ESM.docx]

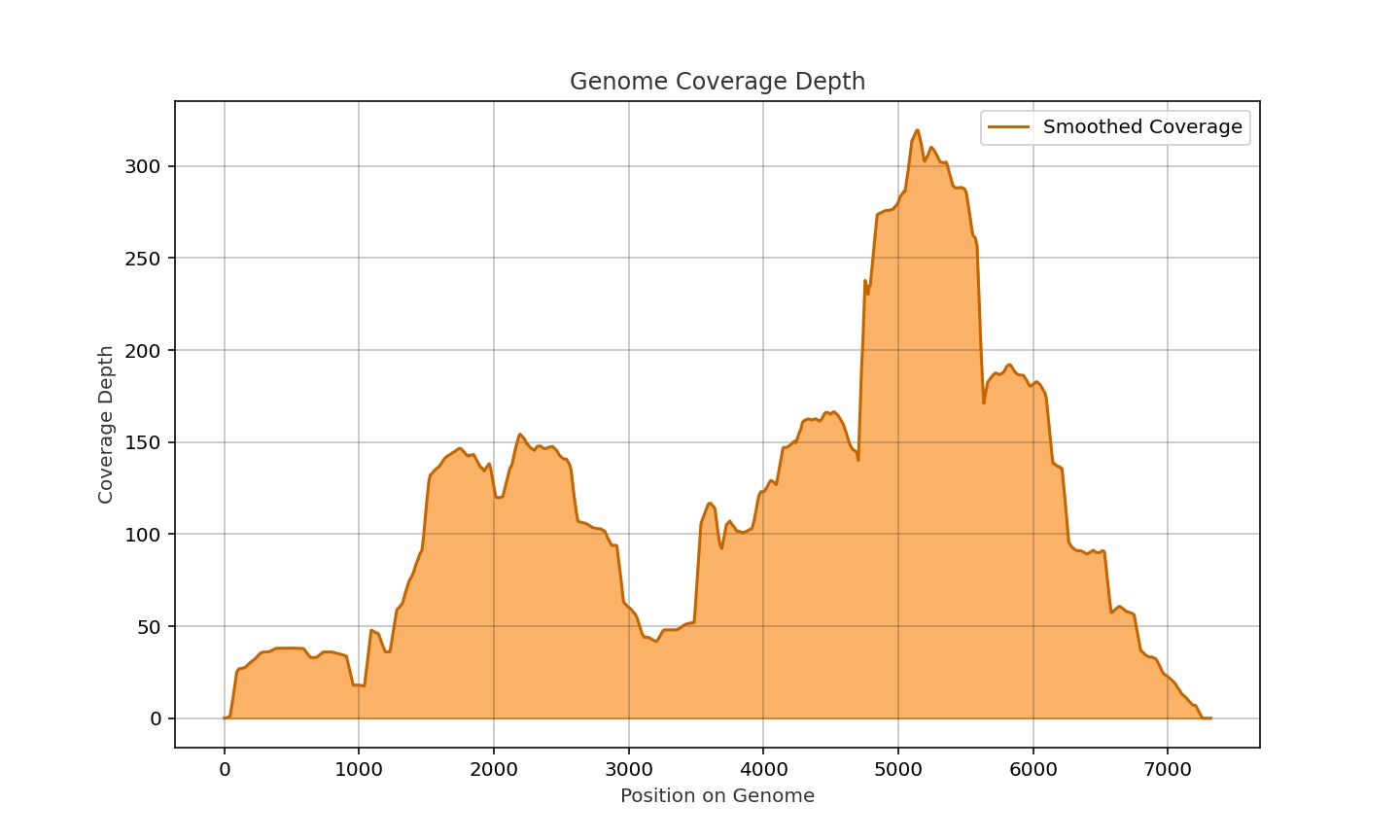


**Supplementary Figure 1.** Coverage depth across the full assembled genome of PV418226 obtained by SISPA amplification and Oxford Nanopore sequencing. The depth of coverage is shown on the y-axis, and the genome position is shown on the x-axis.


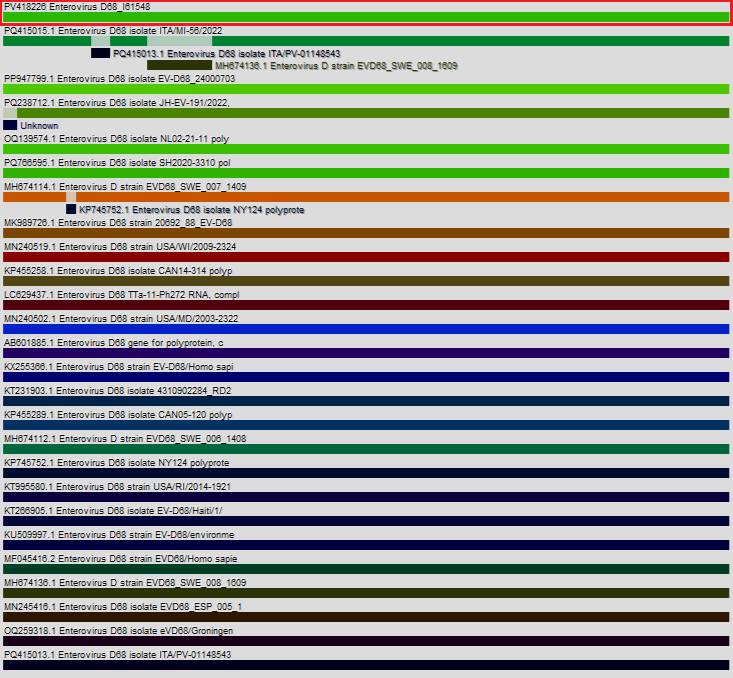


**Supplementary Figure 2.** Recombination analysis of the complete EV-D68 genome (PV418226). Putative recombination events were evaluated using RDP4 with multiple detection methods (RDP, GENECONV, BootScan, MaxChi, Chimaera, SiScan, and 3Seq). The genome recovered in this study is highlighted by a red dashed box. No statistically supported recombination signals were identified for this isolate.


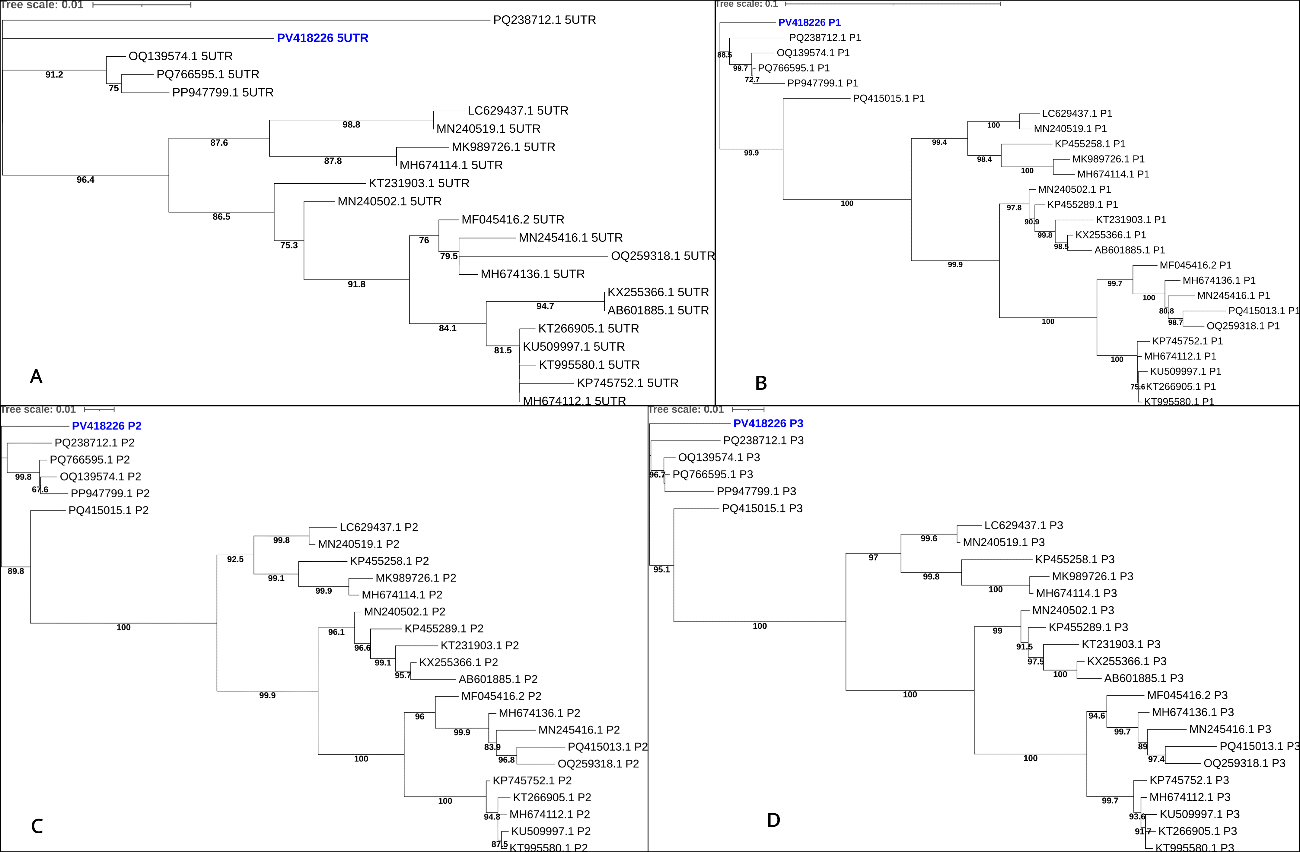


**Supplementary Figure 3.** Maximum-likelihood phylogenetic trees of EV-D68 inferred from different genomic regions of the complete genome recovered in this study. Trees were reconstructed for the 5′UTR (A), P1 region (B), P2 region (C), and P3 region (D) using IQ-TREE under the best-fit substitution model selected for each dataset according to the Bayesian Information Criterion (BIC). Node support values indicate bootstrap percentages based on 1,000 replicates. All available reference sequences included in the dataset at the time of retrieval were analyzed for each genomic region. The genome obtained in this study (PV418226) is highlighted in blue in all trees.


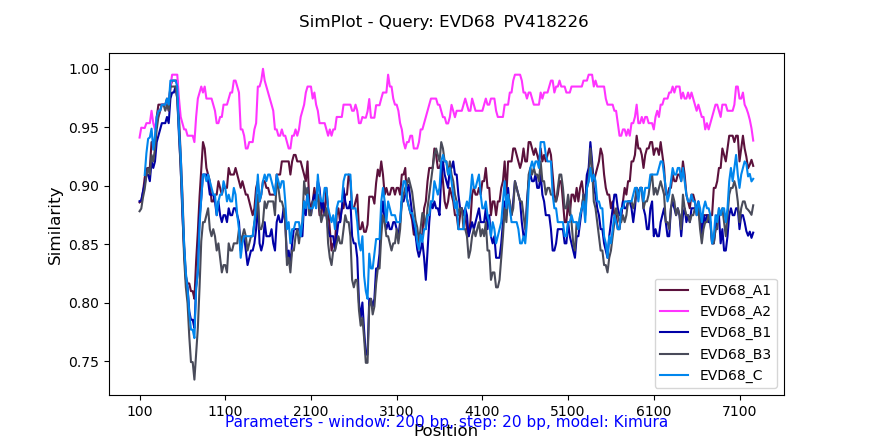


**Supplementary Figure 4.** Similarity plot analysis of the complete EV-D68 genome recovered in this study (PV418226). Nucleotide similarity across the genome was evaluated using SimPlot++ with the study genome as the query sequence and representative EV-D68 reference sequences for clades/subclades A1, A2, B1, B3 and C as comparators. The x-axis indicates genome position and the y-axis shows percentage nucleotide similarity. The analysis showed a consistent similarity profile with clade A2 reference sequences across the genome, with no evidence of mosaic genome structure or recombination.
